# A new species of *Aristotelia* Hübner (Lepidoptera, Gelechiidae, Aristoteliinae) from northern Chile

**DOI:** 10.3897/zookeys.1269.182581

**Published:** 2026-02-13

**Authors:** Héctor A. Vargas

**Affiliations:** 1 Departamento de Recursos Ambientales, Facultad de Ciencias Agronómicas, Universidad de Tarapacá, Arica, Chile Facultad de Ciencias Agronómicas, Universidad de Tarapacá Arica Chile

**Keywords:** Atacama Desert, Andes Mountains, DNA barcoding, micromoth, Neotropical Region, taxonomy

## Abstract

*Aristotelia* Hübner, [1825] (Lepidoptera, Gelechiidae, Aristoteliinae) is a widely distributed moth genus comprising 153 described species, many of which have striking forewings. The variation in genitalia morphology among some members suggests that, as currently circumscribed, the genus is probably not monophyletic. The only Chilean species of *Aristotelia* previously described inhabits the central region of the country. A second Chilean representative, *Aristotelia aguilensis***sp. nov**., is described and illustrated based on adults reared from larvae collected on *Hoffmannseggia minor* (Phil.) Ulibarri (Fabaceae) in the Atacama Desert. The genetic divergence between DNA barcode sequences of the new species was 0.3% (K2P), while it ranged from 11% to 17.1% for other members of the genus. This study reveals a new piece of the *Aristotelia* puzzle, adds a new member to the poorly known gelechiid fauna of northern Chile, and highlights the need to continue fieldwork to reveal the moth diversity that remains overlooked in underexplored Neotropical environments.

## Introduction

The moth subfamily Aristoteliinae (Lepidoptera, Gelechiidae) includes 63 genera (Bidzilya et al. 2025). Although some of these remain poorly known, available taxonomic revisions have shown that genitalia morphology provides important characters for their delimitation (Sattler 1976, 1979; Karsholt and Rutten 2005; Bidzilya and Karsholt 2008, 2021; Huemer and Karsholt 2018). Consequently, examination of the genitalia enables accurate genus assignment for species in this subfamily, including newly discovered ones (Bidzilya and Karsholt 2019; Corley et al. 2025).

The widely distributed genus *Aristotelia* Hübner, [1825], is the most diverse of Aristoteliinae, boasting 153 described species (Hobern et al. 2025), many of which have beautifully colored forewings (Karsholt and Savenkov 2009; Landry and Roque-Albelo 2010; Lee and Brown 2022). The genitalia of *Aristotelia* are characterized by a stout, hook-shaped gnathos, a well-developed sacculus, and short thorns on the phallus in the male, and a ductus bursae with patches of minute spines in the female (Bidzilya and Stonis 2021). However, the variation among some members suggests that, as currently circumscribed, the genus is probably not monophyletic. Previously, a molecular phylogenetic analysis of Gelechiidae that included two representatives of *Aristotelia* (Karsholt et al. 2013) led to the reassignment of one of them to its original genus (Hobern et al. 2025). Furthermore, DNA barcoding has revealed cryptic diversity in both the Old World and the New World (Baer 2018; Huemer et al. 2020). Therefore, a taxonomic revision is required to properly delimit *Aristotelia* and its species (Huemer and Karsholt 2020). Meanwhile, the discovery and description of new species can provide useful information for planning studies that improve understanding of the systematics of this genus and allow for its proper delimitation.

The taxonomy of the gelechiids from northernmost Chile has been little studied, mainly due to the lack of fieldwork and the consequent absence of specimens in scientific collections. Until recently, only a few widely distributed pests were recorded (Cepeda 2017). However, recent searches for larvae associated with native plants have revealed previously unknown native moths of the tribe Gnorimoschemini (Gelechiinae) in the Atacama Desert and the Andes (Vargas 2020, 2025), highlighting the need for additional fieldwork to better understand the diversity of gelechiids inhabiting these arid environments. During these surveys, moths belonging to a new species of *Aristotelia* were reared from larvae collected from the perennial herb *Hoffmannseggia minor* (Phil.) Ulibarri (Fabaceae). Through the taxonomic treatment of this newly discovered moth, this study reveals a new piece of the *Aristotelia* puzzle and adds a new member to the poorly known gelechiid fauna of northern Chile.

## Material and methods

The adult specimens examined in this study were reared from larvae collected on *H. minor* in El Águila (18°29'08"S, 69°51'55"W), at about 1950 m elevation in the Cardones Ravine, Arica Province, northern Chile. The abdomen of each specimen was removed and placed in hot KOH (10%) for a few minutes for dissection of the genitalia, which were then stained with Eosin Y and Chlorazol Black and mounted on slides with Euparal. Photos of the habitus and genitalia were taken using an iPhone 11 camera attached to a Leica M125 stereomicroscope or a Leica DM1000 LED microscope, respectively. The holotype, paratypes, and their genitalia slides are deposited in the “Colección Entomológica de la Universidad de Tarapacá” (IDEA), Arica, Chile.

Genomic DNA was extracted from one pupa and two legs of one adult using the QIAamp Fast DNA Tissue Kit, following the manufacturer’s instructions. DNA purification, PCR amplification, and sequencing of the barcode region (Hebert et al. 2003) with the primers LCO1490 and HCO2198 (Folmer et al. 1994) were performed at Macrogen Inc. (Seoul, South Korea). The PCR program included 5 min at 94 °C, 35 cycles of 30 s at 94 °C, 30 s at 47 °C, 1 min at 72 °C, and a final elongation step of 10 min at 72 °C. The obtained sequences were deposited in the Barcode of Life Data System (**BOLD**) (Ratnasingham and Hebert 2007) under process IDs NCMIC022-25 and NCMIC023-25. MEGA 12 (Kumar et al. 2024) was used to analyze the sequences of the new species with those of other members of *Aristotelia* downloaded from BOLD. Sequence alignment was performed using the ClustalW method, genetic distance was estimated using the Kimura 2-Parameter (**K2P**) method, and a neighbor-joining (**NJ**) tree was constructed based on K2P distances. Branch support of the NJ tree was assessed by 1000 bootstrap replicates.

## Results

### Molecular analysis

**Figure 1.**
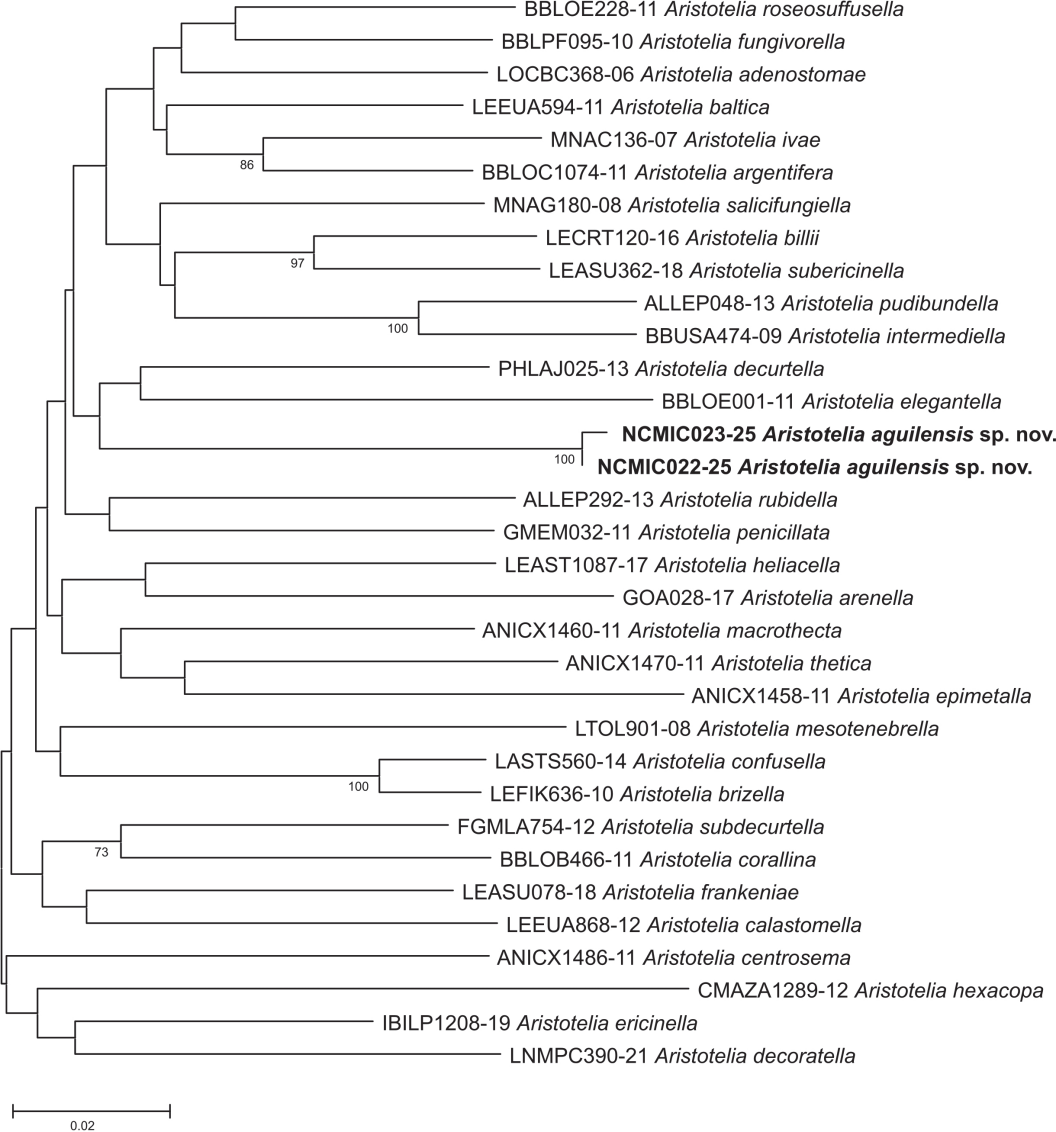
Unrooted neighbor-joining tree of *Aristotelia aguilensis* sp. nov. (bold) and other members of *Aristotelia* Hübner, [1825] (Lepidoptera, Gelechiidae, Aristoteliinae) based on mitochondrial DNA sequences. The BOLD Process ID is recorded to the left of each species name. Numbers indicate bootstrap support (1000 replicates) higher than 70%.

The genetic divergence between the two sequences of the new species was 0.3% (K2P), while it ranged from 11% for *Aristotelia ericinella* (Zeller, 1839) to 17.1% for *Aristotelia hexacopa* Meyrick, 1929 (Fig. 1).

### Taxonomy

#### Aristotelia aguilensis sp. nov.

Taxon classificationAnimaliaOxalidalesElaeocarpaceae

1E0300F6-8B7F-58D7-B7C2-3CDD27CC8F7C

https://zoobank.org/609455F9-F623-4232-96C7-538259CDBD78

Figs 1, 2, 3, 4, 5

##### Type locality.

Chile, Arica Province, Cardones Ravine, El Águila (18°28'40"S, 69°51'38"W), at about 1950 m elevation in the Atacama Desert.

**Figure 2.**
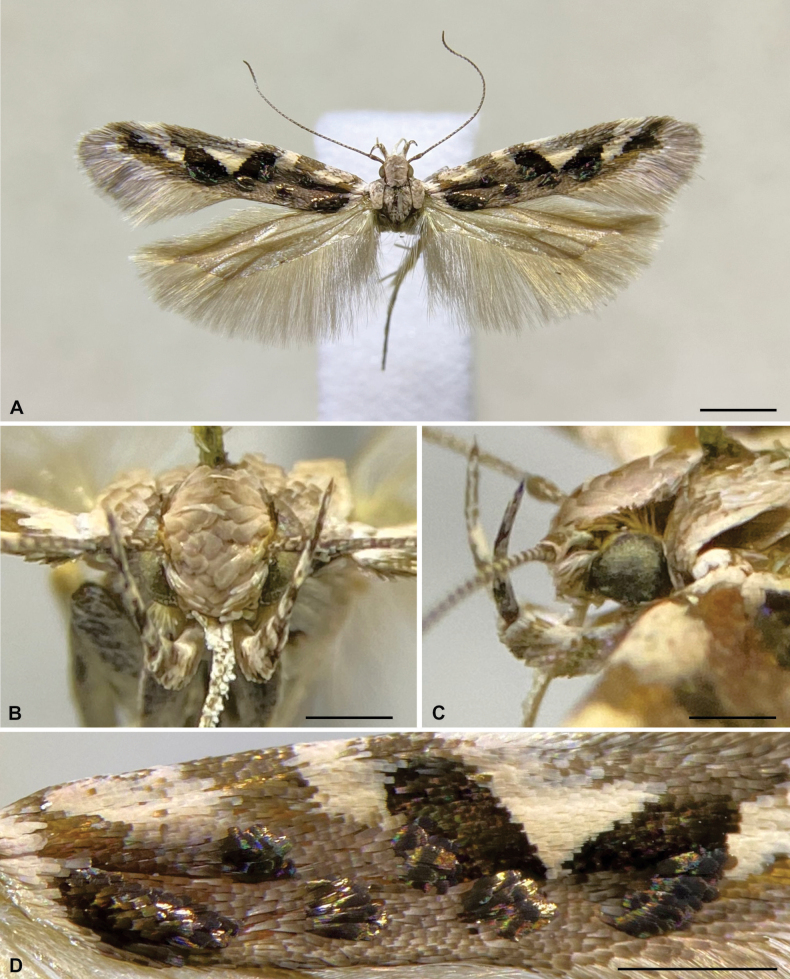
Holotype of *Aristotelia aguilensis* sp. nov. (Lepidoptera, Gelechiidae, Aristoteliinae). **A**. Habitus, dorsal view; **B**. Head, frontal view; **C**. Head, lateral view; **D**. Detail of the right forewing. Scale bars: 2 mm (**A**); 0.5 mm (**B–D**).

**Figure 3.**
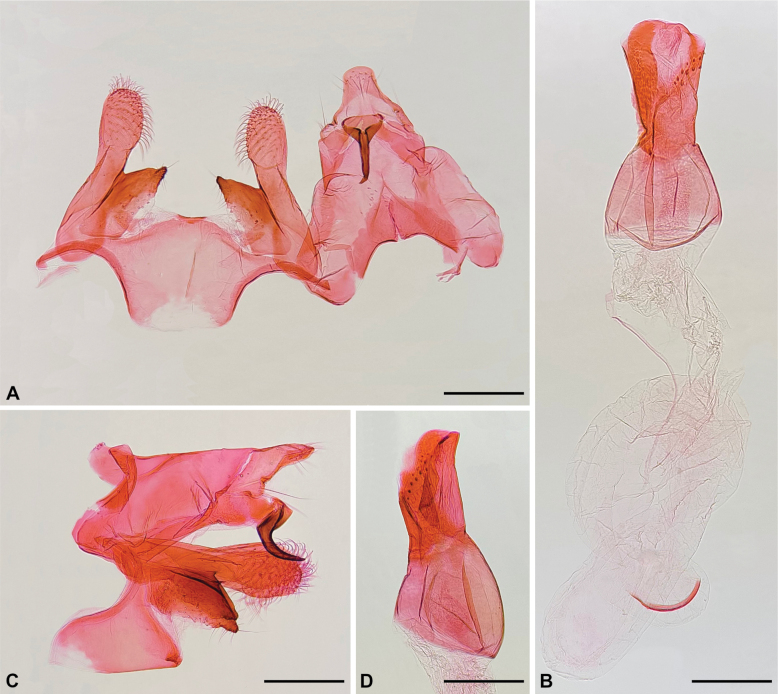
Male genitalia of *Aristotelia aguilensis* sp. nov. (Lepidoptera, Gelechiidae, Aristoteliinae). **A**. Unrolled; left part in dorsal view; right part in ventral view; phallus removed; **B**. Phallus of (**A**), dorsal view; **C**. Lateral view, phallus removed; **D**. Phallus of (**C**), lateral view. Scale bars: 0.3 mm.

**Figure 4.**
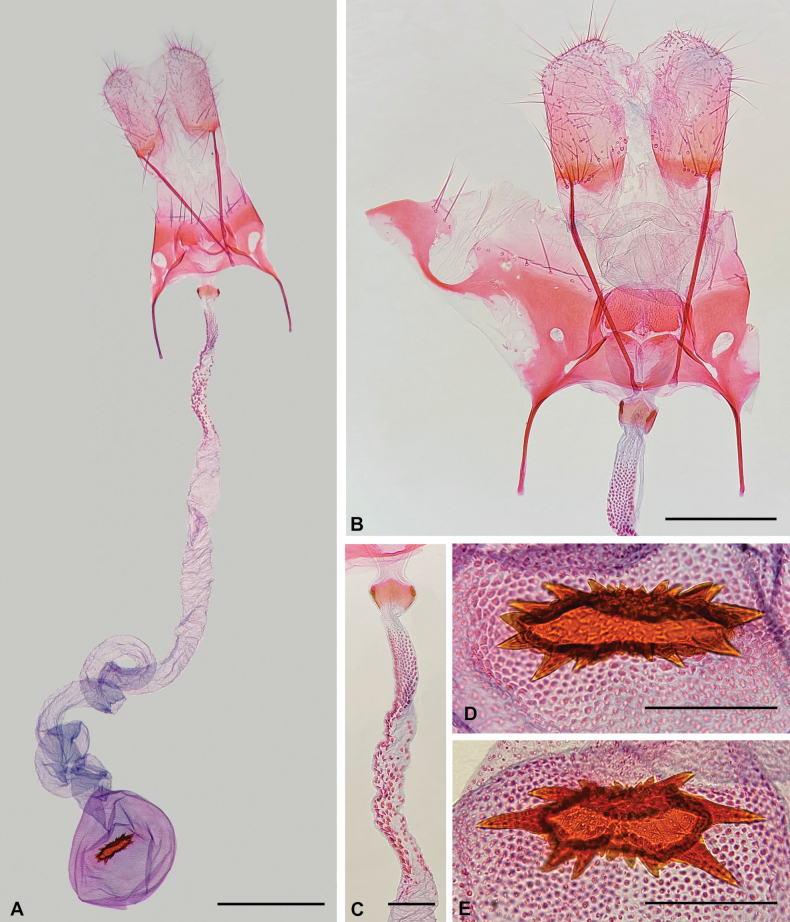
Female genitalia of *Aristotelia aguilensis* sp. nov. (Lepidoptera, Gelechiidae, Aristoteliinae). **A**. Ventral view; **B**. Detail of the posterior part, showing the lamellae connected by narrow elongated longitudinal sclerites, ventral view; **C**. Posterior part of the ductus bursae showing the colliculum and a patch of microtrichia, ventral view; **D, E**. Variation in the shape of the signum, ventral view. Scale bars: 0.5 mm (**A**); 0.3 mm (**B**); 0.1 mm (**C–E**).

**Figure 5.**
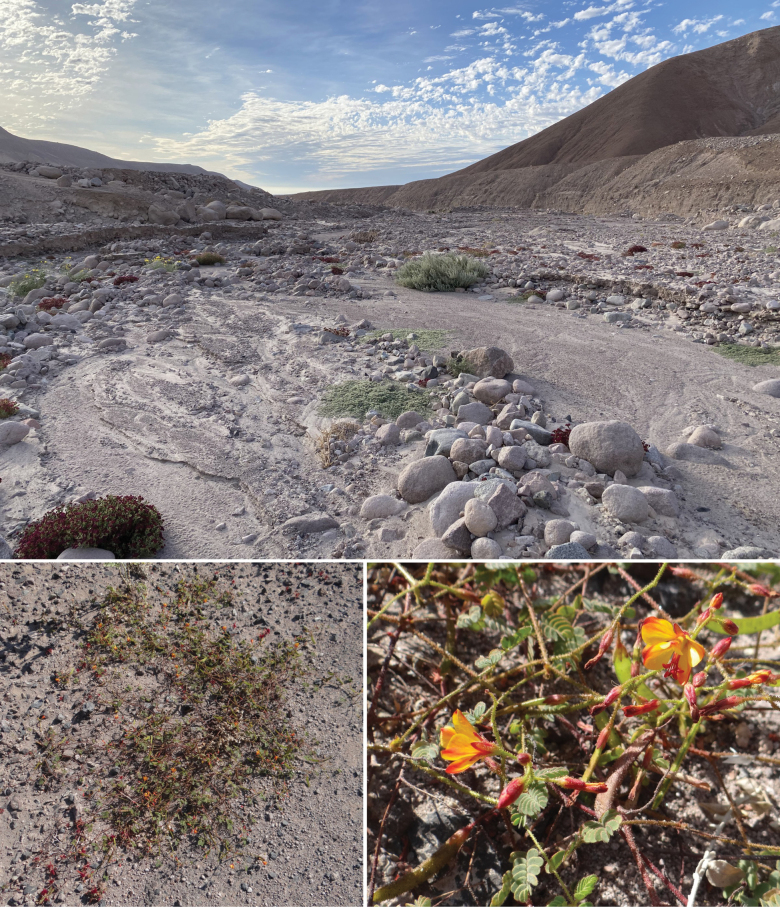
On the natural history of *Aristotelia aguilensis* sp. nov. (Lepidoptera, Gelechiidae, Aristoteliinae) in northern Chile. Above: habitat at the type locality, Cuesta El Águila, Cardones Ravine, Arica Province. Below: the host plant, *Hoffmannseggia minor* (Phil.) Ulibarri (Fabaceae), at the type locality.

##### Type material.

***Holotype***. Chile • ♂; Arica, El Águila; October 2021; H.A. Vargas leg.; ex-larva; *Hoffmannseggia minor*; September 2021; “HOLOTYPE *Aristotelia aguilensis* Vargas” [red handwritten label]; IDEA-LEPI-2025-16; HAV-1656 [genitalia slide]; BOLD accession NCMIC022-25 (IDEA). ***Paratypes***. Chile • 4♂ 5♀; same data as for the holotype; IDEA-LEPI-2025-17 to IDEA-LEPI-2024-25; HAV-1482, 1559, 1560, 1654, 1655, 1657, 1658, 1659, 1875 [genitalia slides] (IDEA).

##### Diagnosis.

*Aristotelia aguilensis* is recognized by its mostly greyish-brown forewings with four creamy-white spots arising from the costa and not reaching the dorsum, the third of which is nearly triangular; and six slightly raised silvery spots, the first of which is a narrow oblique stripe (Fig. 2). Its male genitalia have the distal half of the cucullus free, the dorsal margin of the sacculus fused to the cucullus, the anterior margin of the saccus truncate, and the posterior part of the phallus straight (Fig. 3); and its female genitalia have the lamella postvaginalis as a transverse sub-rectangular plate connected to the lamella antevaginalis by narrow elongated longitudinal sclerites (Fig. 4). Male genitalia of *A. aguilensis* resemble those of *Aristotelia aphiltra* Meyrick, 1917 from central Peru, Ecuador, and Colombia (Meyrick 1917). However, *A. aphiltra* lacks silvery spots on the forewings. Furthermore, the tip of the sacculus does not reach the middle of the cucullus, and the saccus is longer than the cucullus in *A. aphiltra* (Clarke 1969: plate 136, figs 2–2a), while the tip of the sacculus extends beyond the middle of the cucullus, and the saccus is shorter than the cucullus in *A. aguilensis*. The female genitalia of *A. aphiltra* remain unknown, impeding comparisons. *Aristotelia accipiter* Cepeda, 2021 is the only member of the genus previously recorded in Chile; its forewings lack silvery spots, its male genitalia have subtriangular valvae and a sinuous posterior part of the phallus, and its female genitalia lack a lamella postvaginalis (Cepeda 2021: figs 1–11), allowing an accurate separation from *A. aguilensis*.

##### Description.

**Male** (Fig. 2). Head. Vertex mostly covered by broad, flattened, brownish-grey scales with rounded apex, lateral margin with a narrow longitudinal stripe of short, straight, dark brown and yellowish-brown scales. Frons mostly with narrow, flattened, creamy-white scales and scattered yellowish-brown scales. Maxillary palpus creamy white with scattered greyish-brown scales. Labial palpus curved upwards, slightly extending beyond vertex; first segment greyish-brown; second segment mostly creamy white with two ill-defined greyish-brown rings on distal half; third segment mostly creamy white with scattered blackish grey scales on basal half, mostly blackish grey with scattered creamy-white scales on distal half. Haustellum with creamy-white scales. Antenna mostly blackish grey with scattered creamy-white scales. Thorax brownish grey dorsally, creamy white with scattered blackish grey scales laterally. Foreleg with coxa pale pinkish white with scattered blackish grey scales; femur creamy white with scattered blackish grey scales; tibia blackish grey, including epiphysis, with scattered creamy white and pale pinkish white scales; tarsomeres blackish grey with apical creamy-white ring. Midleg coloration similar to that of foreleg, but coxa creamy white, and a pair of tibial spurs creamy white with blackish grey tip. Hindleg creamy white with scattered blackish grey scales on femur; tibia with two blackish grey incomplete rings, two pairs of creamy-white spurs, and a longitudinal row of creamy-white hair-like scales. Forewing (forewing length 7.5–7.8 mm) upper surface mostly greyish brown; a narrow creamy-white basal transverse band; four creamy-white spots of variable size and shape arising from costa but not reaching dorsum, the third one nearly triangular, partially delimited by two broad oblique black spots; a narrow, elongated apical black spot; six slightly raised dark silvery spots, the first a narrow oblique stripe with black inner margin extending from middle of basal transverse band to near dorsal margin, the second at the tip of first creamy-white spot, the third between latter and dorsum, the fourth on inner margin of first black spot, the fifth at tip of first black spot, the sixth along outer margin of second black spot; scattered yellowish scales between second and fourth silvery spots; scattered orange scales between sixth silvery spot and apical black spot; fringe greyish brown, whitish grey and brown. Forewing lower surface greyish brown. Hindwing upper and lower surfaces greyish brown; fringe greyish brown. Abdomen greyish brown. Male genitalia (Fig. 3). Tegumen with broad excavation in the middle of anterior margin. Uncus with short, parallel-sided posterior projection with broadly rounded tip. Gnathos hook-shaped with acute tip, similar in length to uncus. Saccus slightly shorter than central part of tegumen; posterior margin rounded; anterior margin truncate; anteriorly with a non-sclerotized semicircular area. Valva with cucullus elongate, length similar to that of tegumen, rounded tip, not reaching tip of uncus, proximal half fused to sacculus, distal half free, slightly broadened, covered with setae; sacculus rhomboid, about half as long as cucullus, with sparse setae near tip. Phallus about 1.2 times the length of cucullus; anterior half subspherical, strongly swollen; posterior half cylindrical, stout, straight, with a dorsal cleft posteriorly and a double diagonal stripe of small spine-like projections; ductus ejaculatorius about twice length of phallus, with a narrow, slightly curved sclerite about one-third length of phallus.

**Female**. Similar to male in size and maculation. Female genitalia (Fig. 4). Papillae anales rounded, covered with sparse setae. Posterior apophyses rod-shaped, length about 1.6 times the papillae anales. Anterior apophyses rod-shaped, length about 0.4 times the posterior apophyses. Tergum VIII with sinuous anterior margin and a broad circular posterior excavation in the middle; antero-laterally continuous with anterior apophyses and lamella antevaginalis. Lamella antevaginalis a transverse trapezoid-like plate with slightly sinuous anterior margin and straight posterior margin (slightly emarginated in slide-mounted samples), with a triangular non-sclerotized area centrally. Lamella postvaginalis a transverse sub-rectangular plate with microtrichia connected to lamella antevaginalis by narrow elongated longitudinal sclerites. Ductus bursae mostly membranous; colliculum about 1.5 as wide as long; a patch of microtrichia anterior to colliculum, similar in length to posterior apophyses; smaller and denser microtrichia on posterior third of patch; anterior third of ductus bursae coiled. Ductus seminalis arising from side opposite to colliculum. Corpus bursae spherical, diameter similar to posterior apophyses length, mostly membranous; signum ellipsoidal, diameter about one-third that of corpus bursae, with pointed projections of variable size; microtrichia around signum.

##### Etymology.

The specific epithet is derived from the type locality.

##### Distribution and host plant.

(Fig. 5) *Aristotelia aguilensis* has been collected only in the type locality, El Águila, Cardones Ravine, in the Arica Province of northernmost Chile. *Hoffmannseggia minor* is the only host plant known for *A. aguilensis*. This perennial herb occurs in Argentina, Bolivia, and Chile at about 800–4000 m elevation (Rodriguez et al. 2018).

## Discussion

The genitalia morphology of *A. aguilensis* conforms to the general pattern described by Bidzilya and Stonis (2021) for *Aristotelia* by having a stout hook-shaped gnathos, a well-developed sacculus, and short thorns on the phallus in the male, and a ductus bursae with minute spines in the female. Furthermore, *A. aguilensis* matches the character states described by Bidzilya et al. (2025) for *A. decurtella*. However, the new species also exhibits genitalia features that contrast with the latter. The main differences include *A. aguilensis* having a rounded tip of the cucullus without projections, the dorsal margin of the sacculus completely fused to the cucullus, the saccus being as long as it is wide, and the posterior part of the phallus being straight and opening dorsally in the male, and a well-developed lamella postvaginalis connected to the lamella antevaginalis by narrow elongated longitudinal sclerites in the female. In contrast, *A. decurtella* has a cucullus tip with a small spine-like projection, only the proximal half of the sacculus’ dorsal margin fused to the cucullus, the saccus nearly twice as long as it is wide, and the posterior part of the phallus slightly sinuous and opening laterally in the male, and lacks a lamella postvaginalis in the female (https://lepiforum.org/wiki/page/Aristotelia_decurtella; https://pathpiva.fr/aristotelia-decurtella/).

Despite the differences in genitalia morphology from the type species, assigning the new species to *Aristotelia* seems reasonable until there is a better delimitation of this genus and a more detailed understanding of the South American fauna of Aristoteliinae. Like previous studies (Baer 2018; Metz et al. 2019), the discovery of *A. aguilensis* represents a good example of the challenges that can be found among the little-known Neotropical gelechiids and highlights the need for continued fieldwork to reveal the moth diversity that remains overlooked in underexplored Neotropical environments.

## Supplementary Material

XML Treatment for Aristotelia aguilensis

## Additional information

### Use of AI

No use of AI was reported.

### Author ORCIDs

Héctor A. Vargas https://orcid.org/0000-0002-5355-3157

### Data availability

All of the data that support the findings of this study are available in the main text.
